# Characterization of linearly graded position-sensitive silicon photomultipliers

**DOI:** 10.1186/2197-7364-1-S1-A14

**Published:** 2014-07-29

**Authors:** Alessandro Ferri, Fabio Acerbi, Alberto Gola, Giovanni Paternoster, Claudio Piemonte, Nicola Zorzi

**Affiliations:** Fondazione Bruno Kessler, Trento, Italy

We present the characterization of a novel Position-Sensitive Silicon Photomultiplier, called Linearly-Graded SiPM (LG-SiPM). The position encoding is obtained through a charge sharing approach, implemented by means of a current divider, directly integrated on die.

We fabricated prototypes of the 2D LG-SiPM with two different dimensions, with an active area of 8x8 mm^2^ and 4x4 mm^2^. The microcells of the detectors have a size of 45x45 μm^2^ and they are fabricated with the FBK RGB-SiPM technology. The technology was modified with the addition of a double metal interconnection layer and a second quenching resistor, required by the 2D LG-SiPM architecture.

We measured the intrinsic spatial resolution of a 4x4 mm^2^ LG-SiPM by illuminating it with short (~30 ns long) LED pulses in different positions, spaced by 0.25 mm. The peak wavelength of the LED was 410 nm. The illumination spot had a diameter of 1 mm, which covers approximately 330 microcells and simulates the light emitted by a pixelated LYSO with 1 mm pitch. The peaks are easily separated, indicating an intrinsic spatial resolution well below 0.25 mm. Additionally, no pincushion distortion is present.

We characterized the detector with a 4x4x10 mm^3^ fine pixelated (0.8 mm pitch) LYSO matrix without optical isolation between pixels, with 511 keV gamma rays to verify the encoding capability in PET conditions. Despite the substantial amount of light sharing introduced by the scintillator, we were able to easily distinguish the crystal elements. Measurements with a pixelated CsI(Tl) in SPECT conditions (i.e., with 122 keV gamma) are ongoing and will be presented in the poster.

